# Tetra­kis(picolinato-κ^2^ *N*,*O*)zirconium(IV) dihydrate

**DOI:** 10.1107/S1600536811031710

**Published:** 2011-08-17

**Authors:** Maryke Steyn, Hendrik G. Visser, Andreas Roodt, T. J. Muller

**Affiliations:** a Department of Chemistry, University of the Free State, PO Box 339, Bloemfontein 9300, South Africa

## Abstract

In the title compound, [Zr(C_6_H_4_NO_2_)_4_]·2H_2_O, the Zr^IV^ atom is located on a crystallographic fourfold rotoinversion axis (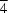) and is coordinated by four picolinate anions with Zr—O and Zr—N distances of 2.120 (2) and 2.393 (2) Å, respectively. An approximate square-anti­prismatic coordination polyhedron of the *N*,*O*-coordination ligand atoms is formed, with a distortion towards dodeca­hedral geometry. The crystal packing is stabilized by inter­molecular π–π inter­actions between adjacent picolinate rings [centroid–centroid distances = 3.271 (1) and 3.640 (2) Å], as well as O—H⋯O hydrogen bonds between the solvent mol­ecules and the coordinated ligands, thereby linking the mol­ecules into a supra­molecular three-dimensional network.

## Related literature

For *N*,*O*- and *O*,*O*′-bidentate ligand complexes of zirconium and hafnium, see: Steyn *et al.* (2008 ▶); Viljoen *et al.* (2010**a* ▶,b* ▶). For relevant studies of *N*,*O*- and *O*,*O*′-bidentate ligands with other transition metal atoms, see: Graham *et al.* (1991 ▶); Mtshali *et al.* (2006 ▶); Roodt *et al.* (2011 ▶); Schutte *et al.* (2008 ▶); Steyn *et al.* (1997 ▶); Van Aswegen *et al.* (1991 ▶); Van der Westhuizen *et al.* (2010 ▶). 
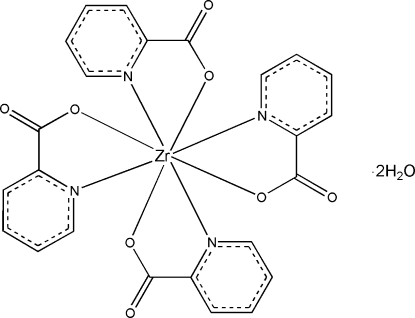


## Experimental

### 

#### Crystal data

[Zr(C_6_H_4_NO_2_)_4_]·2H_2_O
*M* *_r_* = 615.66
Tetragonal, 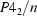

*a* = 11.083 (5) Å
*c* = 9.548 (5) Å
*V* = 1172.8 (10) Å^3^
*Z* = 2
Mo *K*α radiation
μ = 0.54 mm^−1^
*T* = 100 K
0.12 × 0.09 × 0.04 mm

#### Data collection

Bruker X8 APEXII 4K Kappa CCD diffractometer
Absorption correction: multi-scan (*SADABS*; Bruker, 2004 ▶) *T*_min_ = 0.942, *T*_max_ = 0.977
27234 measured reflections
1477 independent reflections
1271 reflections with *I* > 2σ(*I*)
*R*_int_ = 0.074

#### Refinement

*R*[*F* ^2^ > 2σ(*F* ^2^)] = 0.037
*wR*(*F* ^2^) = 0.100
*S* = 1.10
1477 reflections
87 parameters
1 restraint
H atoms treated by a mixture of independent and constrained refinement
Δρ_max_ = 0.64 e Å^−3^
Δρ_min_ = −0.92 e Å^−3^

### 

Data collection: *APEX2* (Bruker, 2010 ▶); cell refinement: *SAINT-Plus* (Bruker, 2004 ▶); data reduction: *SAINT-Plus*; program(s) used to solve structure: *SHELXS97* (Sheldrick, 2008 ▶); program(s) used to refine structure: *SHELXL97* (Sheldrick, 2008 ▶); molecular graphics: *DIAMOND* (Brandenburg, 2006 ▶); software used to prepare material for publication: *WinGX* (Farrugia, 1999 ▶).

## Supplementary Material

Crystal structure: contains datablock(s) global, I. DOI: 10.1107/S1600536811031710/zq2119sup1.cif

Structure factors: contains datablock(s) I. DOI: 10.1107/S1600536811031710/zq2119Isup2.hkl

Additional supplementary materials:  crystallographic information; 3D view; checkCIF report

## Figures and Tables

**Table 1:** Hydrogen-bond geometry (Å, °)

*D*—H⋯*A*	*D*—H	H⋯*A*	*D*⋯*A*	*D*—H⋯*A*
O03—H03*A*⋯O2^i^	0.94 (2)	1.89 (2)	2.829 (3)	175 (5)

Symmetry code: (i) 
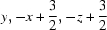
.

**Fig. 1.**
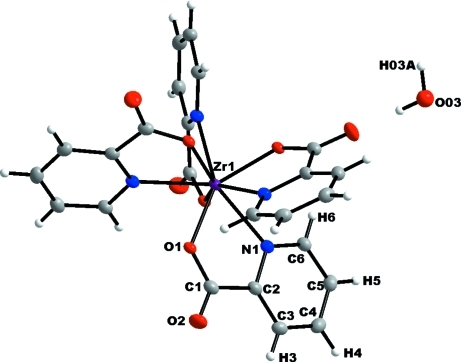
Representation of the title compound with displacement ellipsoids drawn at the 50% probability level. H atoms are presented as small spheres of arbitrary radius.

**Fig. 2.**
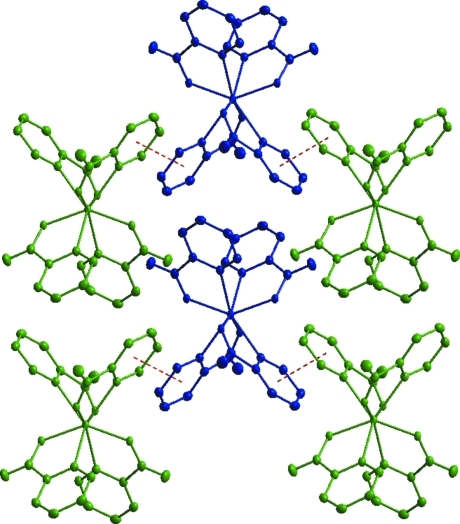
Graphical illustration of π–π interaction and stacking between different PicA-ligands of neighboring molecules to form a three-dimensional network (displacement ellipsoids are drawn at the 50% probability level). Hydrogen atoms and solvent water molecules omitted for clarity.

**Fig. 3.**
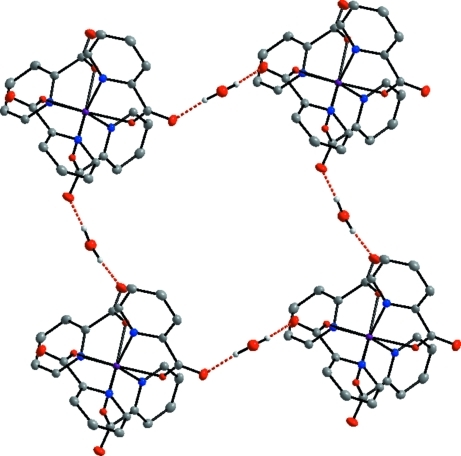
Graphical illustration of Zr(PicA)4 indicating O–H···O hydrogen bonding interaction as observed between the solvent molecules and the free carbonyl oxygen atoms from neighboring molecules (displacement ellipsoids are drawn at the 50% probability level).

## supplementary crystallographic information

### Comment

The introduction of N,*O*-bidentate ligands with the oxine or aminovinylketone backbones significantly influences both steric and electronic properties of transition metal centres as illustrated by literature examples (Graham *et al.*, 1991; Mtshali *et al.*, 2006; Roodt *et al.*, 2011; Schutte *et al.*, 2008; Steyn *et al.*, 1997; Van Aswegen *et al.*, 1991; Van der Westhuizen *et al.*, 2010). This study is part of ongoing research initiatives investigating coordination behaviour of *O*,*O*'- and *N*, *O*,-bidentate ligands with zirconium(IV) and hafnium(IV) for possible separation of these two metals from base ore sources (Steyn *et al.*, 2008; Viljoen *et al.*, 2010*a*,*b*).

The title compound, [Zr(C_6_H_4_NO_2_)_4_].2H_2_O, with C_6_H_4_NO_2_ as picolinic acid, crystallizes in the form of colourless cubic crystals in the tetragonal P4_2_/n space group. The Zr^IV^ atom, located on a crystallographic fourfold rotoinversion axis (4), is coordinated to four picolinic acid ligands (Fig. 1). The assymetric unit contains half a solvent molecule located on a twofold axis. The Zr—O and Zr—N bond lengths are 2.120 (2) Å and 2.393 (2) Å, respectively, with a N—Zr—O bite angle of 69.79 (7) °. The coordination polyhedron around the metal centre is an approximate square antiprism of the N,*O*-coordination ligand atoms, with a distortion towards dodecahedral geometry. The crystal packing is stabilized by intermolecular π-π interactions (Fig. 2), between adjacent picolinato rings, with interplanar and centroid-to-centroid distances of 3.271 (1) Å and 3.640 (2) Å, respectively. Further stabilization of the crystal structure is afforded by O—H···O hydrogen bonding (Fig. 3) between the carbonyl group of the picolinato ligands and the solvent water molecules. All of these interactions serve to link the molecules into a supramolecular three-dimensional network.

### Experimental

Chemicals were purchased from Sigma-Aldrich and used as received. ZrCl_4_ (103.3 mg, 0.463 mmol) and picolinic acid (PicA) (175.2 mg, 1.423 mmol) was separately dissolved in DMF (2.5 ml ea) and heated to 60 °C. The PicA solution was added drop-wise to the zirconium solution and stirred at 60 °C for 30 minutes. The reaction solution was removed from heating, covered and left to stand for crystallization. White cubic crystals, suitable for single-crystal X-ray diffraction, formed after 30 days (yield: 178 mg, 86%).

### Refinement

The aromatic H atoms were placed in geometrically idealized positions (C–H = 0.95 Å) and constrained to ride on their parent atoms with *U*_iso_(H) = 1.2*U*_eq_(C). The hydrogen atoms of the solvent water molecule were located on the Fourier difference map and refined isotropically. The highest residual electron density was located 0.74 Å from O1.

### Crystal data

**Table:**

[Zr(C_6_H_4_NO_2_)_4_]·2H_2_O	*D*_x_ = 1.743 Mg m^−^^3^
*M**_r_* = 615.66	Mo *K*α radiation, λ = 0.71073 Å
Tetragonal, *P*4_2_/*n*	Cell parameters from 9933 reflections
Hall symbol: -P 4bc	θ = 2.6–28.4°
*a* = 11.083 (5) Å	µ = 0.54 mm^−^^1^
*c* = 9.548 (5) Å	*T* = 100 K
*V* = 1172.8 (10) Å^3^	Cuboid, colourless
*Z* = 2	0.12 × 0.09 × 0.04 mm
*F*(000) = 624	

### Data collection

**Table:**

Bruker X8 APEXII 4K Kappa CCD diffractometer	1477 independent reflections
Radiation source: fine-focus sealed tube	1271 reflections with *I* > 2σ(*I*)
graphite	*R*_int_ = 0.074
ω and φ scans	θ_max_ = 28.5°, θ_min_ = 2.6°
Absorption correction: multi-scan (*SADABS*; Bruker, 2004)	*h* = −14→13
*T*_min_ = 0.942, *T*_max_ = 0.977	*k* = −14→14
27234 measured reflections	*l* = −12→12

### Refinement

**Table:**

Refinement on *F*^2^	Primary atom site location: structure-invariant direct methods
Least-squares matrix: full	Secondary atom site location: difference Fourier map
*R*[*F*^2^ > 2σ(*F*^2^)] = 0.037	Hydrogen site location: inferred from neighbouring sites
*wR*(*F*^2^) = 0.100	H atoms treated by a mixture of independent and constrained refinement
*S* = 1.10	*w* = 1/[σ^2^(*F*_o_^2^) + (0.0415*P*)^2^ + 2.5407*P*] where *P* = (*F*_o_^2^ + 2*F*_c_^2^)/3
1477 reflections	(Δ/σ)_max_ < 0.001
87 parameters	Δρ_max_ = 0.64 e Å^−^^3^
1 restraint	Δρ_min_ = −0.92 e Å^−^^3^

### Special details

**Table:**

Experimental. The intensity data were collected on a Bruker X8 ApexII 4 K Kappa CCD diffractometer using an exposure time of 40 s/frame. A total of 1709 frames were collected with a frame width of 0.5° covering up to θ = 28.40° with 99.5% completeness accomplished.
Geometry. All s.u.'s (except the s.u. in the dihedral angle between two l.s. planes) are estimated using the full covariance matrix. The cell s.u.'s are taken into account individually in the estimation of s.u.'s in distances, angles and torsion angles; correlations between s.u.'s in cell parameters are only used when they are defined by crystal symmetry. An approximate (isotropic) treatment of cell s.u.'s is used for estimating s.u.'s involving l.s. planes.
Refinement. Refinement of *F*^2^ against ALL reflections. The weighted *R*-factor *wR* and goodness of fit *S* are based on *F*^2^, conventional *R*-factors *R* are based on *F*, with *F* set to zero for negative *F*^2^. The threshold expression of *F*^2^ > 2σ(*F*^2^) is used only for calculating *R*-factors(gt) *etc*. and is not relevant to the choice of reflections for refinement. *R*-factors based on *F*^2^ are statistically about twice as large as those based on *F*, and *R*- factors based on ALL data will be even larger.

### Fractional atomic coordinates and isotropic or equivalent isotropic displacement parameters (Å^2^)

**Table:**

	*x*	*y*	*z*	*U*_iso_*/*U*_eq_	
Zr1	0.25	0.25	0.75	0.01305 (15)	
O1	0.41797 (14)	0.31522 (14)	0.82454 (18)	0.01305 (15)	
O2	0.56146 (18)	0.33223 (18)	0.9859 (2)	0.0263 (4)	
C3	0.4700 (2)	0.1154 (2)	1.1212 (3)	0.0195 (5)	
H3	0.5415	0.144	1.1601	0.023*	
N1	0.31289 (18)	0.13671 (18)	0.9505 (2)	0.0157 (4)	
C2	0.4174 (2)	0.1737 (2)	1.0089 (3)	0.0169 (5)	
C5	0.3088 (2)	−0.0267 (2)	1.1131 (3)	0.0206 (5)	
H5	0.2707	−0.0959	1.1461	0.025*	
C6	0.2603 (2)	0.0372 (2)	1.0016 (3)	0.0178 (5)	
H6	0.1891	0.01	0.961	0.021*	
C4	0.4143 (2)	0.0137 (2)	1.1748 (3)	0.0222 (5)	
H4	0.4473	−0.0269	1.251	0.027*	
C1	0.4728 (2)	0.2822 (2)	0.9379 (3)	0.0183 (5)	
O03	0.25	0.75	0.3385 (4)	0.0472 (9)	
H03A	0.274 (4)	0.815 (3)	0.396 (4)	0.068 (15)*	

### Atomic displacement parameters (Å^2^)

**Table:**

	*U*^11^	*U*^22^	*U*^33^	*U*^12^	*U*^13^	*U*^23^
Zr1	0.01054 (17)	0.01054 (17)	0.0181 (2)	0	0	0
O1	0.01054 (17)	0.01054 (17)	0.0181 (2)	0	0	0
O2	0.0201 (9)	0.0257 (10)	0.0332 (11)	−0.0070 (8)	−0.0066 (8)	0.0008 (8)
C3	0.0170 (11)	0.0211 (12)	0.0205 (12)	0.0015 (9)	−0.0014 (9)	−0.0035 (10)
N1	0.0141 (9)	0.0136 (9)	0.0194 (10)	−0.0005 (8)	0.0000 (8)	−0.0002 (8)
C2	0.0145 (11)	0.0160 (11)	0.0203 (12)	0.0000 (9)	0.0007 (9)	−0.0030 (9)
C5	0.0221 (12)	0.0178 (12)	0.0220 (13)	0.0026 (9)	0.0043 (10)	0.0027 (10)
C6	0.0158 (11)	0.0154 (11)	0.0223 (12)	−0.0003 (9)	0.0005 (9)	0.0003 (9)
C4	0.0236 (13)	0.0240 (13)	0.0191 (13)	0.0056 (10)	0.0003 (10)	0.0020 (10)
C1	0.0148 (11)	0.0171 (11)	0.0231 (12)	−0.0001 (9)	0.0006 (9)	−0.0032 (9)
O03	0.059 (2)	0.038 (2)	0.045 (2)	−0.0011 (18)	0	0

### Geometric parameters (Å, °)

**Table:**

Zr1—O1^i^	2.1200 (18)	C3—C4	1.384 (4)
Zr1—O1	2.1200 (18)	C3—H3	0.93
Zr1—O1^ii^	2.1200 (18)	N1—C6	1.340 (3)
Zr1—O1^iii^	2.1200 (18)	N1—C2	1.349 (3)
Zr1—N1^i^	2.393 (2)	C2—C1	1.511 (4)
Zr1—N1^ii^	2.393 (2)	C5—C4	1.384 (4)
Zr1—N1^iii^	2.393 (2)	C5—C6	1.386 (4)
Zr1—N1	2.393 (2)	C5—H5	0.93
O1—C1	1.294 (3)	C6—H6	0.93
O2—C1	1.218 (3)	C4—H4	0.93
C3—C2	1.381 (4)	O03—H03A	0.941 (19)
			
O1^i^—Zr1—O1	96.47 (3)	N1^i^—Zr1—N1	129.78 (7)
O1^i^—Zr1—O1^ii^	96.47 (3)	N1^ii^—Zr1—N1	73.76 (11)
O1—Zr1—O1^ii^	140.77 (10)	N1^iii^—Zr1—N1	129.78 (7)
O1^i^—Zr1—O1^iii^	140.77 (10)	C1—O1—Zr1	126.61 (15)
O1—Zr1—O1^iii^	96.47 (3)	C2—C3—C4	118.7 (2)
O1^ii^—Zr1—O1^iii^	96.47 (3)	C2—C3—H3	120.7
O1^i^—Zr1—N1^i^	69.79 (7)	C4—C3—H3	120.7
O1—Zr1—N1^i^	145.95 (7)	C6—N1—C2	118.2 (2)
O1^ii^—Zr1—N1^i^	73.05 (7)	C6—N1—Zr1	126.65 (17)
O1^iii^—Zr1—N1^i^	78.95 (7)	C2—N1—Zr1	114.94 (16)
O1^i^—Zr1—N1^ii^	145.95 (7)	N1—C2—C3	122.8 (2)
O1—Zr1—N1^ii^	78.95 (7)	N1—C2—C1	113.9 (2)
O1^ii^—Zr1—N1^ii^	69.79 (7)	C3—C2—C1	123.3 (2)
O1^iii^—Zr1—N1^ii^	73.05 (7)	C4—C5—C6	119.3 (2)
N1^i^—Zr1—N1^ii^	129.78 (7)	C4—C5—H5	120.4
O1^i^—Zr1—N1^iii^	78.95 (7)	C6—C5—H5	120.4
O1—Zr1—N1^iii^	73.05 (7)	N1—C6—C5	122.1 (2)
O1^ii^—Zr1—N1^iii^	145.95 (7)	N1—C6—H6	118.9
O1^iii^—Zr1—N1^iii^	69.79 (7)	C5—C6—H6	118.9
N1^i^—Zr1—N1^iii^	73.76 (11)	C3—C4—C5	118.9 (2)
N1^ii^—Zr1—N1^iii^	129.78 (7)	C3—C4—H4	120.6
O1^i^—Zr1—N1	73.05 (7)	C5—C4—H4	120.6
O1—Zr1—N1	69.79 (7)	O2—C1—O1	124.4 (2)
O1^ii^—Zr1—N1	78.95 (7)	O2—C1—C2	121.4 (2)
O1^iii^—Zr1—N1	145.95 (7)	O1—C1—C2	114.2 (2)

Symmetry codes: (i) *y*, −*x*+1/2, −*z*+3/2; (ii) −*x*+1/2, −*y*+1/2, *z*; (iii) −*y*+1/2, *x*, −*z*+3/2.

### Hydrogen-bond geometry (Å, °)

**Table:**

*D*—H···*A*	*D*—H	H···*A*	*D*···*A*	*D*—H···*A*
O03—H03A···O2^iv^	0.94 (2)	1.89 (2)	2.829 (3)	175 (5)

Symmetry codes: (iv) *y*, −*x*+3/2, −*z*+3/2.
